# Hemi-ECMO: A Novel Method of Left Ventricular Afterload Reduction for Venoarterial Extracorporeal Membrane Oxygenation (VA-ECMO)

**DOI:** 10.3390/bioengineering13050499

**Published:** 2026-04-24

**Authors:** Christian Said, Christopher Hayward, Michael Stevens, Gabriel Matus Vazquez, Laurence Boss, Ricardo Deveza, Sumita Barua, Kavitha Muthiah, Pankaj Jain

**Affiliations:** 1 School of Medicine, The University of New South Wales, Kensington 2052, Australia; 2 The Victor Chang Cardiac Research Institute, Darlinghurst 2010, Australia; 3 Department of Cardiology, St Vincent’s Hospital, Darlinghurst 2010, Australia; 4 Department of Cardiology, Royal Prince Alfred Hospital, Camperdown 2050, Australia; 5 School of Medicine, The University of Sydney, Camperdown 2050, Australia

**Keywords:** left ventricle, unloading, mechanical circulatory support, mock circulatory loop, biventricular, cardiogenic shock, aortic occlusion

## Abstract

Venoarterial extracorporeal membrane oxygenation (VA-ECMO) has failed to demonstrate mortality benefit in randomised controlled trials of cardiogenic shock. We aimed to determine whether a novel ‘Hemi-ECMO’ configuration, involving aortic occlusion to isolate the left ventricle from the VA-ECMO circuit, improves cardiac haemodynamics. We utilised a pulsatile biventricular mock circulatory loop with variable contractility to compare standard VA-ECMO with Hemi-ECMO support under left ventricular or biventricular failure conditions. When averaged across all pump speeds, mean left atrial pressure was significantly reduced with Hemi-ECMO compared to VA-ECMO (21.11 ± 1.32 mmHg vs. 26.53 ± 0.87 mmHg, *p* < 0.001), with more pronounced benefit at higher pump speeds. Aortic ejection increased with Hemi-ECMO at higher pump speeds: 0.14 ± 0.03 vs. 0.00 ± 0.00 L/min (*p* = 0.002) at 3000 revolutions per minute (RPM). Aortic ejection was greater with Hemi-ECMO in the descending aorta compared to the ascending aorta position (0.27 ± 0.03 L/min vs. 0.17 ± 0.05 L/min, *p* = 0.015). In conclusion, Hemi-ECMO demonstrates significant haemodynamic advantages in severe cardiogenic shock, including reductions in mean left atrial pressure and increases in aortic ejection, with greater benefits when positioned in the descending aorta. Further in vivo studies are warranted to assess clinical viability.

## 1. Introduction

Venoarterial extracorporeal membrane oxygenation (VA-ECMO) is increasingly utilised for patients experiencing severe cardiogenic shock [1,2,3]. Although VA-ECMO effectively provides full circulatory support and restores systemic perfusion, it has failed to demonstrate mortality benefit in randomised controlled trials of cardiogenic shock [4,5]. This failure may be due to VA-ECMO’s adverse haemodynamic and cardiac effects. VA-ECMO increases systemic afterload, which can detrimentally impact the function of an impaired left ventricle (LV) by reducing left ventricular stroke volume [6] and increased LV filling pressure [7,8,9]. In severe cases, complete cessation of aortic ejection leads to left ventricular distension [7,8], pulmonary oedema [7], and thrombus formation [9], a catastrophic complication.

Current strategies to address these issues include pharmacological reduction in systemic afterload, augmentation of contractility, and reduction in VA-ECMO pump support [10]. However, these approaches have significant limitations, particularly in hypotensive patients where end-organ perfusion may be further compromised and in patients with severely impaired myocardial function [11,12]. In these cases, the only remaining clinical strategy involves the introduction of additional mechanical circulatory support devices to directly decompress or ‘unload’ the left ventricle, most commonly using transvalvular microaxial flow pumps [13,14,15,16,17,18]. While effective, microaxial flow pumps are associated with high complication rates [19], significant cost, and the need for complex and specialised care to manage two separate mechanical support devices. A recent large multicentre randomised controlled trial of microaxial flow pump use during infarct-related cardiogenic shock demonstrated a significantly higher rate of device-related safety events (24.0% vs. 6.2%) including severe bleeding, limb ischaemia requiring intervention, and renal replacement therapy (41.9% vs. 26.7%) compared to usual care [19]. Simpler, safer, and lower-cost solutions are needed [16,20,21].

Previous studies have demonstrated that the deleterious haemodynamic effects of VA-ECMO are likely to be pressure-mediated, rather than flow-mediated [10,11,12]. In severe LV dysfunction, the systemic arterial pressure required to adequately perfuse vital organs may exceed the pressure against which the LV can effectively eject. We therefore postulate that creating a pressure gradient between the LV and the remainder of the vital organs may provide a solution to this problem.

**Figure 1:**
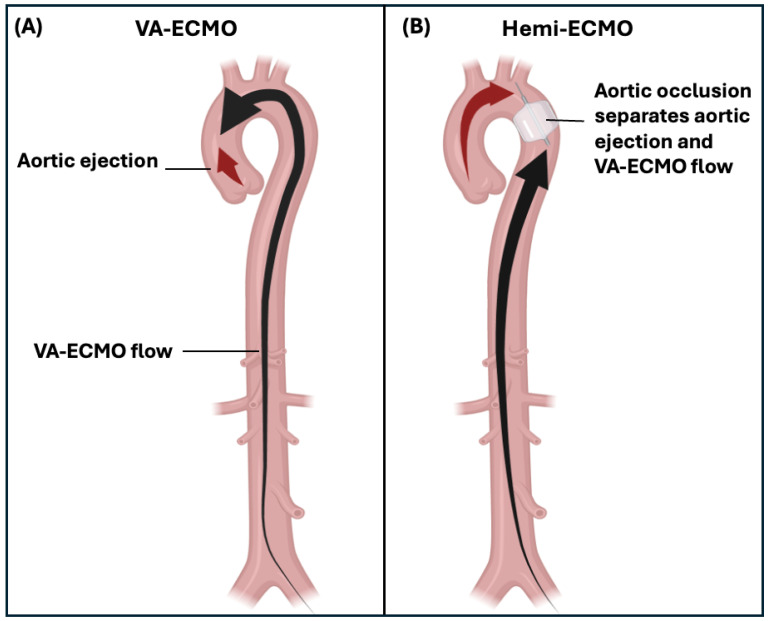
The Hemi-ECMO concept. (**A**) Peripheral VA-ECMO: Retrograde arterial flow from the VA-ECMO circuit increases systemic afterload against which the impaired left ventricle must eject (black arrows indicate direction of blood flow). (**B**) Hemi-ECMO: An occlusive balloon in the aorta isolates the left ventricle from VA-ECMO-generated afterload, creating two separate circulations. The native left ventricle supplies the circulation proximal to the occlusive balloon via aortic ejection (red arrow) while VA-ECMO perfuses the systemic circulation distal to the occlusive balloon. Abbreviations: VA-ECMO, venoarterial extracorporeal membrane oxygenation; LV, left ventricle.

We propose a novel strategy, ‘Hemi-ECMO’, which involves obstructing the aorta to completely isolate the left ventricle from the VA-ECMO circuit, thereby creating two separate ‘hemi’ circulations. Unlike existing unloading strategies that decompress the LV by draining volume or intraventricular pressure, Hemi-ECMO’s principle is unique in aiming to directly reduce the afterload experienced by the LV. We postulate that by decoupling LV afterload from systemic perfusion pressure, the LV may eject into a low-resistance proximal circulation while VA-ECMO supplies the systemic circulation distal to the aortic occlusion (Figure 1). Using a biventricular mock circulatory loop (BVMCL) [22], we aimed to: (1) determine the effects of Hemi-ECMO on left atrial pressure and aortic ejection; and (2) determine the optimal position of occlusion for Hemi-ECMO, in terms of the same haemodynamic parameters.

## 2. Materials and Methods

**Figure 2:**
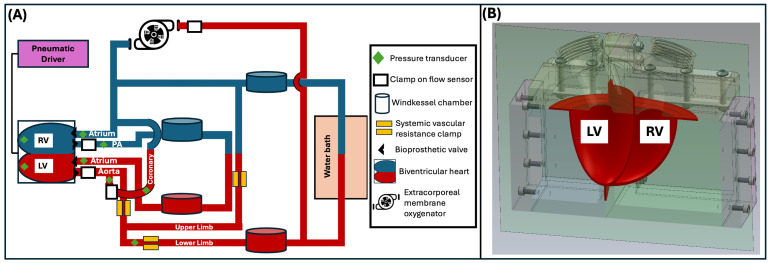
Biventricular mock loop (BVMCL). (**A**) Circuit schematic of the BVMCL incorporating venoarterial extracorporeal membrane oxygenation, coronary artery and upper limb circulations. Sensor locations (pressure transducers, flow probes) and key components are indicated in the legend. Red tubing denotes arterial circulation; blue denotes venous return. (**B**) Cut-away rendering of the 3D-printed biventricular heart and enclosure. Abbreviations: BVMCL, biventricular mock circulatory loop; LV, left ventricle; PA, pulmonary artery; RV, right ventricle.

We utilised a previously validated, pulsatile biventricular mock circulatory loop (BVMCL) [22], which incorporates a Frank–Starling mechanism, interventricular septal interaction, and four bioprosthetic valves in anatomically correct positions. The BVMCL was modified to incorporate a coronary and upper limb circulation representing 5% and 20% of cardiac output respectively (Figure 2A). The BVMCL consisted of 3D-printed components (Figure 2B) including pneumatically driven left and right silicone ventricles with a common interventricular septum. Four 25 mm porcine surgical bioprosthetic valves were seated in the anatomically correct positions. Systemic and pulmonary circuits were constructed from Tygon E-3603 tubing incorporating acrylic Windkessel compliance chambers. Flow was measured using calibrated Transonic clamp-on ultrasonic flow probes (Transonic Systems, Ithaca, NY, USA), and pressures were recorded with fluid-filled transducers and Millar (Millar, Houston, TX, USA) Mikro-Tip catheters. A programmable controller drove the ventricles pneumatically and implemented a Frank–Starling relationship by adjusting LV contractility (end-systolic pressure–volume relationship) in response to changes in mean left atrial pressure [22].

A Maquet (Maquet Cardiopulmonary, Rastatt, Germany) Rotaflow^®^ VA-ECMO system was integrated into the lower limb and right atrium of the BVMCL along with a Maquet^®^ 17Fr arterial cannula and Maquet 25FR venous cannula. The circuit was filled with 40% *w*/*w* glycerol solution heated to 35 °C (±2 °C) via an inline water bath, a Maquet HU 35^®^ Heater Unit and Maquet Quadrox iD heat exchanger corresponding to a haematocrit of 35%. To achieve the effect of an occlusive balloon in the aorta, a VA-ECMO tubing clamp was applied in one of 2 positions: the first occlusive position was in the ascending aorta, distal to the take-off of the coronary artery circuit but proximal to the upper limb circulation; the second position was in the descending aorta, distal to the origin of the upper limb circulation.

A left ventricular failure state (LVF) was induced with the following haemodynamic parameters: mean aortic pressure 50 mmHg (±5 mmHg), mean left atrial pressure 28 mmHg (±2 mmHg), right atrial pressure 15 mmHg (±2 mmHg), and cardiac output 1.6 L/min (±0.1 L/min). Once the LVF state was stable, the ascending aorta was completely occluded and 30 s recordings were obtained at 100 Hz. The system was then returned to baseline LVF conditions without occlusion, after which the descending aorta was occluded and recordings were repeated. This experimental sequence was repeated three times at each VA-ECMO pump speed—0 RPM (pump off), 2000, 2500, 3000, 3500, and 4000 RPM—spanning the clinical operating range of VA-ECMO pumps and corresponding to partial through full circulatory support. The entire experimental protocol was then repeated under biventricular failure (BVF) conditions with mean aortic pressure 50 mmHg (±5 mmHg), mean left atrial pressure 25 mmHg (±2 mmHg), right atrial pressure 20 mmHg (±2 mmHg), and cardiac output 1.6 L/min (±0.1 L/min). Failure states were induced by reducing pneumatic drive pressure to the respective ventricles. Both isolated LVF and BVF were studied as they represent the two most common phenotypes of cardiogenic shock requiring VA-ECMO support.

We assessed the effect of Hemi-ECMO on the following outcomes: mean left atrial pressure (mLAP), measured via a fluid-filled pressure transducer in the left atrium; aortic ejection, defined as forward flow across the aortic valve generated by the native left ventricle; pump flow, defined as flow generated by the VA-ECMO centrifugal pump; and coronary artery flow. All flows were measured using calibrated clamp-on ultrasonic flow probes (Transonic Systems, Ithica, NY, USA). 

Each experimental condition at each VA-ECMO pump speed was repeated across 2 heart failure phenotypes, 2 occlusion positions, and 3 replicates, yielding *n* = 12 per pump speed. Data were analysed using paired *t*-tests with MATLAB R2024b (MathWorks, MA, USA) and RStudio version 2025.05.1 (Posit, MA, USA). Results are reported as means ± standard error of the mean (SEM) and *p*-values to three decimal places.

## 3. Results

Complete aortic occlusion was achieved in all experiments, generating a mean trans-occlusive pressure gradient of 58.85 mmHg ± 7.16 mmHg (*p* < 0.001). The mean pressure gradient was 45.79 ± 8.99 mmHg for ascending aorta occlusion (*p* < 0.001) and 71.74 ± 10.79 mmHg for descending aorta occlusion (*p* < 0.001).

### 3.1. Mean Left Atrial Pressure

**Figure 3:**
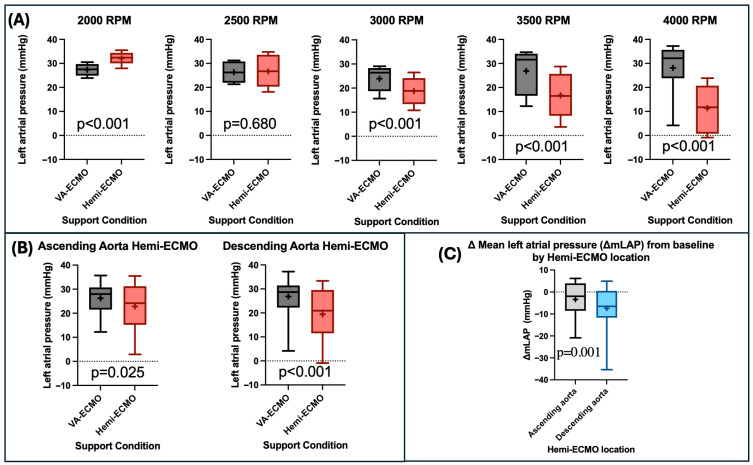
Changes in mean left atrial pressure (mLAP) with Hemi-ECMO. (**A**) Comparison of mLAP between VA-ECMO and Hemi-ECMO at varying RPM levels. (**B**) Comparison of mLAP between VA-ECMO and Hemi-ECMO in both the ascending aortic and descending aortic positions. (**C**) Comparison of the delta mean left atrial pressure (ΔmLAP) from baseline of Hemi-ECMO in the ascending aortic and descending aortic positions. Abbreviations: RPM—revolutions per minute; ΔmLAP—delta mean left atrial pressure. Legend: “+”—statistical mean.

Mean left atrial pressure (mLAP, averaged across all pump speeds and occlusion positions) was significantly lower with Hemi-ECMO than with VA-ECMO (21.11 ± 1.32 mmHg vs. 26.53 ± 0.87 mmHg, *p* < 0.001). This effect was more pronounced at higher VA-ECMO support levels (Table 1) (Figure 3A). At 2000 RPM, mLAP was higher with Hemi-ECMO (*p* < 0.001) and there was no significant change in mLAP between Hemi-ECMO and VA-ECMO at 2500 RPM (Table 1).

Hemi-ECMO significantly reduced mLAP in both the ascending (22.80 ± 1.76 mmHg vs. 26.21 ± 1.65 mmHg, *p* = 0.025) and descending (19.49 ± 1.96 mmHg vs. 26.84 ± 1.32 mmHg, *p* < 0.001) positions (Figure 3B). The reduction in mLAP from baseline was greater in the descending position compared to ascending Hemi-ECMO (7.42 ± 1.77 mmHg vs. 3.41 ± 1.45 mmHg, *p* = 0.001) (Figure 3C). Additionally, the reduction in mLAP was greater in isolated LV failure compared to biventricular failure (6.69 ± 1.89 mmHg vs. 4.14 ± 1.34 mmHg, *p* = 0.044).

### 3.2. Aortic Ejection

**Figure 4:**
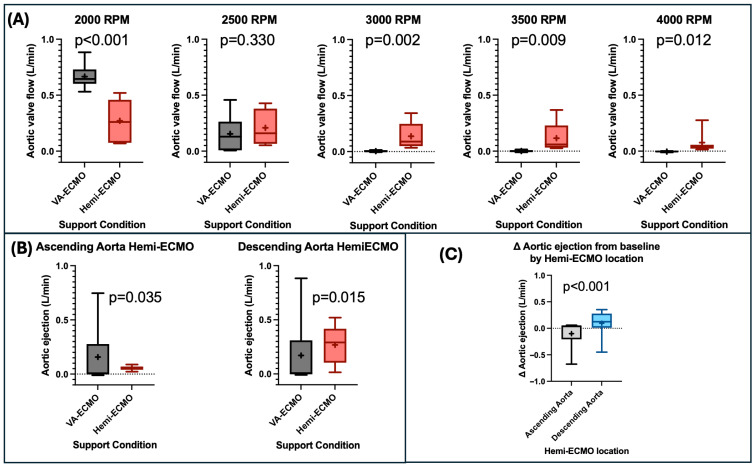
Changes in aortic ejection with Hemi-ECMO. (**A**) Comparison of aortic ejection between VA-ECMO and Hemi-ECMO at varying RPM levels. (**B**) Comparison of aortic ejection between VA-ECMO and Hemi-ECMO in both the ascending aortic and descending aortic positions. (**C**) Comparison of the delta aortic ejection from baseline of Hemi-ECMO in the ascending aortic and descending aortic positions. Abbreviations: RPM—revolutions per minute. Legend: “+”—statistical mean.

When aortic ejection was assessed in both Hemi-ECMO positions and across all pump speeds combined, there was no difference between Hemi-ECMO and traditional VA-ECMO (0.16 ± 0.02 L/min vs. 0.16 ± 0.035 L/min, *p* = 0.95). When analysed by pump speed, aortic ejection was significantly higher with Hemi-ECMO at pump speeds above 2500 RPM (Table 1). No significant difference in aortic ejection was observed between Hemi-ECMO and VA ECMO at 2500 RPM (Table 1). Aortic ejection was reduced by Hemi-ECMO at 2000 RPM (*p* < 0.001) (Table 1) (Figure 4A).

When analysed across all VA-ECMO pump speeds combined, aortic ejection increased with descending aorta Hemi-ECMO (0.27 ± 0.03 L/min vs. 0.17 ± 0.05 L/min, *p* = 0.015) but decreased with ascending aorta Hemi-ECMO (0.06 ± 0.00 L/min vs. 0.16 ± 0.04 L/min, *p* = 0.035) (Figure 4B). The change in aortic ejection from baseline was significantly greater with descending compared to ascending aorta Hemi-ECMO (0.10 ± 0.04 L/min vs. −0.10 ± 0.05 L/min, *p* < 0.001) (Figure 4C). Nevertheless, both ascending and descending aorta Hemi-ECMO maintained some degree of aortic ejection at all VA-ECMO speeds, whereas VA-ECMO alone caused cessation of aortic ejection at VA-ECMO speeds of 3000 RPM and higher. No difference in the change in aortic ejection from baseline was noted between isolated left ventricular failure and biventricular failure (*p* = 0.987).

### 3.3. Pump Flow

Hemi-ECMO resulted in a statistically significant decrease in pump flow compared to VA-ECMO when averaged across all pump speeds (2.57 ± 0.10 L/min vs. 2.64 ± 0.11 L/min, *p* < 0.001). The pump flow with Hemi-ECMO was significantly lower at >2000 RPM, while at 2000 RPM, Hemi-ECMO produced higher pump flow than VA-ECMO (Table 1).

Pump flow was significantly lower with descending aorta Hemi-ECMO (2.48 ± 0.14 L/min vs. 2.64 ± 0.16 L/min, *p* < 0.001) but was not significantly changed with ascending aorta Hemi-ECMO (2.61 ± 0.14 L/min vs. 2.64 ± 0.16 L/min, *p* = 0.210). The reduction in pump flow from baseline was greater with descending aorta Hemi-ECMO compared to ascending aorta Hemi-ECMO (0.16 ± 0.03 L/min vs. 0.04 ± 0.03 L/min, *p* < 0.001). The reduction in pump flow from baseline was greater in isolated left ventricular failure compared to biventricular failure (0.11 ± 0.03 vs. 0.03 ±0.02 L/min, *p* < 0.001).

### 3.4. Coronary Artery Flow

**Figure 5:**
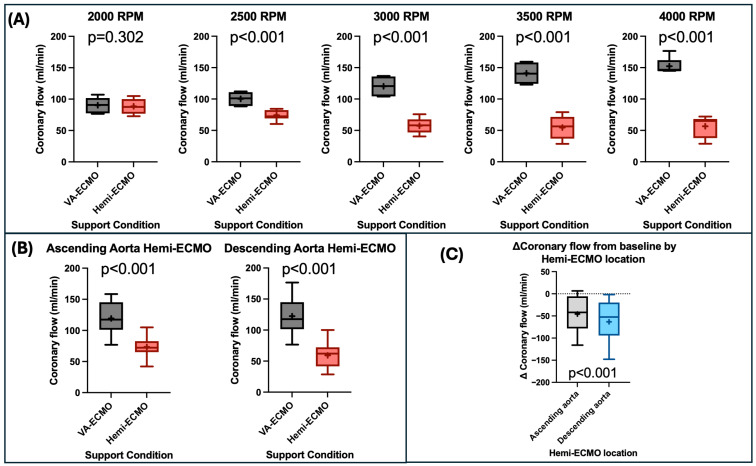
Changes in coronary artery flow with Hemi-ECMO. (**A**) Comparison of coronary artery flow between VA-ECMO and Hemi-ECMO at varying RPM levels. (**B**) Comparison of coronary artery flow between VA-ECMO and Hemi-ECMO in both the ascending aortic and descending aortic positions. (**C**) Comparison of the delta coronary artery flow from baseline of Hemi-ECMO in the ascending aortic and descending aortic positions. Abbreviations: RPM—revolutions per minute. Legend: “+”—statistical mean.

Coronary artery flow was decreased with Hemi-ECMO compared to VA-ECMO (66.30 ± 2.41 mL/min vs. 120.80 ± 3.55 mL/min, *p* < 0.001), with greater differences at higher RPMs. Coronary artery flow was lower with Hemi-ECMO between 4000 and 2500 RPM inclusive (all *p* < 0.001) (Table 1) (Figure 5A). Coronary flow was not significantly different with Hemi-ECMO at 2000 RPM (Table 1) (Figure 5A).

There was no significant difference in coronary flow between the baseline cardiogenic shock state (without VA-ECMO) and ascending aorta Hemi-ECMO at 4000 RPM (72.98 ± 4.68 mL/min vs. 68.48 ± 1.29 mL/min, *p* = 0.410). Coronary flow was decreased with descending aorta Hemi-ECMO at 4000 RPM compared to the baseline cardiogenic shock state without VA-ECMO (44.79 ± 6.71 mL/min vs. 75.82 ± 5.02 mL/min, *p* = 0.042).

Hemi-ECMO significantly reduced coronary artery flow in both the ascending aorta (73.00 ± 2.70 mL/min vs. 119.00 ± 4.70 mL/min, *p* < 0.001) and descending aorta (59.00 ± 3.60 mL/min vs. 122.00 ± 3.60 mL/min, *p* < 0.001) positions (Figure 5B). The reduction in coronary flow from baseline was significantly greater with descending vs. ascending aorta Hemi-ECMO (63.00 ± 8.50 mL/min vs. 46.00 ± 6.90 mL/min, *p* < 0.001) (Figure 5C). Furthermore, the reduction in coronary flow from baseline was greater in isolated left ventricular failure compared to biventricular failure (76.00 ± 9.20 mL/min vs. 38.00 ± 5.60 mL/min, *p* < 0.001). There was a linear correlation between coronary flow and mLAP with Hemi-ECMO (r^2^ = 0.66, *p* < 0.001).

## 4. Discussion

Although the use of VA-ECMO is steadily increasing, randomised controlled trials continue to demonstrate a lack of mortality benefit [4,5], highlighting the need for strategies to mitigate VA-ECMO’s potential adverse effects including aortic valve non-ejection, left ventricular distension, and increased left atrial pressure. Current approaches to these issues are costly, invasive, and can carry significant risk of complications [13,17].

We developed and assessed the effectiveness of ‘Hemi-ECMO’, a novel aortic occlusion technique during VA-ECMO. Our main findings include the following: (1) Hemi-ECMO decreased mLAP; (2) descending aorta Hemi-ECMO reduced mLAP to a greater degree than ascending aorta positioning; (3) descending aorta Hemi-ECMO increased aortic valve ejection; and (4) Hemi-ECMO decreased coronary artery flow.

### 4.1. Hemi-ECMO Decreases Mean Left Atrial Pressure

Mean left atrial pressure (mLAP) is closely associated with left ventricular end-diastolic pressure [23,24] and pulmonary capillary wedge pressure [25,26]. Under normal conditions, mLAP ranges between 2 and 12 mmHg [24], while elevations >25 mmHg are strongly linked to the development of pulmonary oedema [27], a common indication for left ventricular decompression [28,29]. Hemi-ECMO effectively lowered mLAP, with a mean reduction of 5.42 mmHg (*p* < 0.001) compared to VA-ECMO alone. The reduction was more pronounced at higher levels of VA-ECMO support, reaching a mean difference of 16.80 mmHg at 4000 RPM (*p* < 0.001). These findings suggest that Hemi-ECMO may help mitigate pulmonary oedema risk in VA-ECMO supported patients and, critically, provides the greatest benefit at higher pump speeds where it is most likely to be needed. Clinically, this degree of LAP reduction may be sufficient to alleviate pulmonary congestion and reduce the need for mechanical LV decompression which remains a leading indication for adjunctive device therapy during VA-ECMO [13,14,15].

We hypothesise that this effect is driven by three concurrent mechanisms. First, with Hemi-ECMO, the left ventricle is shielded from the afterload generated by VA-ECMO, leading to improved native cardiac output. Second, as the venous arm of VA-ECMO continues to drain the right atrium, there is reduced inflow to the right ventricle, resulting in decreased transpulmonary flow and therefore reduced left ventricular preload. Third, reduced right-sided filling pressure is directly transmitted to the left side of the heart via the interventricular and interatrial septa.

Notably, at the lowest pump speed tested (2000 RPM), mLAP was higher with Hemi-ECMO than with VA-ECMO alone. We hypothesise that at low pump speeds with Hemi-ECMO, venous drainage is insufficient to reduce left ventricular preload. This may result in a net increase in LV filling pressure. These findings underscore that the haemodynamic benefits of Hemi-ECMO may be dependent on adequate VA-ECMO pump speeds and pre-load reduction.

### 4.2. Descending Aorta Hemi-ECMO Produces Greater Reduction in mLAP

Descending aorta Hemi-ECMO produced a greater reduction in mLAP compared to the ascending aortic position. The descending aorta position likely provides a lower impedance circulation with a greater vascular territory into which the LV can eject, compared to the ascending position in which LV ejection is limited to the coronary circulation alone. This result indicates a potentially greater cardioprotective effect with more distal positioning of the occlusive balloon and may enable device delivery via the left radial approach.

Ascending aorta Hemi-ECMO avoids the concern of cerebral ischaemia, as VA-ECMO continues to perfuse all vessels distal to the coronary arteries. In contrast, descending aorta Hemi-ECMO relies on the native heart to perfuse the cerebral circulation. If the native heart fails to generate sufficient oxygenated cardiac output, widespread cerebral ischaemia may occur. To mitigate the effect of isolating the bilateral common carotid and vertebral arteries from VA-ECMO, the ideal placement of a Hemi-ECMO aortic balloon occlusion may be between the right brachiocephalic and left common carotid arteries, permitting VA-ECMO to perfuse the entire cerebral circulation via the left common carotid and subclavian arteries and an intact Circle of Willis. Monitoring of regional cerebral perfusion could be performed using near-infrared spectroscopy (NIRS). The impact of descending aorta Hemi-ECMO on cerebral perfusion is a critical unanswered question, and future large animal studies will be essential to establish the viability of this technique.

### 4.3. Hemi-ECMO Increases Aortic Ejection

Reduced aortic valve ejection can lead to ventricular distension [7,30], and thrombus formation [31]. Furthermore, existing forms of ‘venting’, including surgical vents, do not solve the problem of non-ejection. The only clinically available solution that simultaneously reduces LV filling pressure while increasing aortic ejection is placement of an additional mechanical circulatory support device such as a transvalvular microaxial flow pump.

Hemi-ECMO significantly increases aortic valve flow compared to VA-ECMO alone at higher, clinically relevant levels of VA-ECMO support (Figure 4A). This effect is most prominent when Hemi-ECMO is positioned in the descending aorta, likely due to the increased pressure gradient across the occlusion in this position compared to the ascending aorta. Hemi-ECMO may therefore provide a simple, low-cost solution to this potentially catastrophic and often intractable complication.

We hypothesise that the modest reduction in VA-ECMO pump flow observed with Hemi-ECMO (0.07 L/min, ~2.7%) (Table 1) is unlikely to be clinically significant in isolation. With Hemi-ECMO, total systemic perfusion represents the combined output of VA-ECMO pump flow to the circulation distal to the occlusion and native left ventricular ejection to the proximal circulation. As Hemi-ECMO preserves aortic ejection at pump speeds where VA-ECMO alone results in complete cessation, the net effect on total systemic perfusion may be neutral or favourable. Finally, the vascular territory supplied by the VA-ECMO pump during Hemi-ECMO is smaller, and therefore may require less flow to maintain adequate perfusion.

### 4.4. Hemi-ECMO Decreases Combined Coronary Artery Flow

Our finding of a reduction in coronary flow with Hemi-ECMO is predictable, as the Hemi-ECMO occlusion prevents VA-ECMO flow from reaching the coronary circulation. Additionally, reduced LV filling pressure with Hemi-ECMO results in reduced LV contraction via the Frank–Starling mechanism, meaning that native LV output is unable to compensate fully for this loss of VA-ECMO supply. A strong correlation was observed between decreased mean left atrial pressure (mLAP) and coronary flow, supporting this hypothesis.

It remains unclear whether this reduction in coronary flow would necessarily result in myocardial ischaemia. Our model incorporated a simplified coronary circulation comprising a single outflow representing 5% of cardiac output, and it is uncertain whether the native coronary circulation, with distinct left and right coronary artery territories and autoregulation, would behave similarly. Hemi-ECMO effectively reduces LV pressure and volume, and therefore pressure–volume area (PVA). Furthermore, PVA correlates linearly with myocardial oxygen consumption (VO_2_) [32,33,34,35,36] such that reductions in PVA correspond to decreased myocardial metabolic demand and a lower requirement for coronary flow. We hypothesise that by isolating the left ventricle from the afterload imposed by VA-ECMO, Hemi-ECMO may allow for systemic vasodilator therapy to enhance coronary flow while also permitting high VA-ECMO pump speeds to maintain adequate blood pressure and systemic perfusion. Whether the observed reduction in coronary flow translates to myocardial ischaemia in vivo remains an important unanswered question, and its clinical significance must be carefully evaluated in large animal studies.

### 4.5. Limitations

Our study was conducted in vitro using a BVMCL, which has inherent limitations, particularly its inability to fully replicate the dynamic interactions between the heart and circulation. The BVMCL does not completely capture the complex interplay of the heart, vasculature, and neurohormonal responses seen in clinical cardiogenic shock. We partially mitigated this through incorporation of a Frank–Starling mechanism and interventricular interactions, the combination of which has not, to our knowledge, been incorporated into mock circulatory loops elsewhere. Nevertheless, coronary, cerebral, and renal autoregulation and neurohumoral responses are absent from our BVMCL and may modulate these findings in vivo.

Patients on VA-ECMO support experience continuous haemodynamic fluctuations due to factors such as pharmacological titration and changes in intravascular volume, whereas our experiments were performed under controlled, steady-state conditions.

Vascular and bleeding events continue to be common adverse effects of VA-ECMO therapy in cardiogenic shock [4,5,29,37]. Our 40% *w*/*w* aqueous-glycerol solution is well validated [38,39] and routinely used in mock circulatory loops [40,41,42,43,44,45,46]. While 40% *w*/*w* aqueous glycerol serves as a reasonable substitute given that human blood behaves as a Newtonian fluid in large arteries such as the aorta [47], it does not fully replicate all blood’s properties, including its non-Newtonian behaviour in smaller vessels [39,47] and coagulation.

An important unanswered question with the Hemi-ECMO technique is its propensity to cause or promote arterial thrombosis. Additionally, aortic occlusion may carry inherent risks including aortic injury, impaired myocardial perfusion, distal organ ischaemia, and haemodynamic decompensation from rapid afterload change during balloon deflation. These risks require evaluation in large animal studies.

Given these limitations, our results are hypothesis-generating, and raise a number of questions to be answered in future large animal studies and clinical research.

### 4.6. Future Directions and Clinical Translation

In vivo studies are required to resolve several potential issues in the application of Hemi-ECMO. These studies would aim to confirm the effectiveness of the Hemi-ECMO technique, assess for complications including thrombus formation, assess the effect on cerebral perfusion, and quantify the effects on myocardial function, blood flow and oxygen demand.

If shown to be safe and effective in vivo, we anticipate the clinical implementation of the Hemi-ECMO technique using a balloon positioned in the aortic arch, deployable via radial or femoral arterial access similar to an intra-aortic balloon pump. Notably, the concept of aortic balloon tamponade of the descending aorta has been applied successfully in the setting of haemorrhagic shock [48,49,50].

Should in vivo studies confirm these findings, Hemi-ECMO may offer several advantages over microaxial flow pumps and surgical left ventricular decompression. We anticipate a Hemi-ECMO balloon would be significantly easier to insert and manage compared to a transvalvular microaxial flow pump, whilst potentially being substantially more cost-effective. This would have the advantage of being more widely available for use in patients with VA-ECMO and may enhance accessibility and broaden its clinical application in VA-ECMO-supported patients, particularly in healthcare systems with financial constraint. Additionally, Hemi-ECMO’s anticipated smaller bore access and potential for radial delivery could mitigate many of the vascular complications associated with surgical and percutaneous left ventricular decompression devices.

## 5. Conclusions

Assessed in a biventricular mock circulatory loop, ‘Hemi-ECMO’, a novel method of afterload reduction during venoarterial extracorporeal membrane oxygenation, demonstrates significant haemodynamic advantages in vitro, including a significant reduction in mean left atrial pressure and an increase in aortic ejection when positioned in the descending aorta. These findings warrant further evaluation in large animal models.

## Figures and Tables

**Table 1:** Hemodynamic parameters during VA-ECMO and Hemi-ECMO support by pump speed. Values are mean ± SEM from paired 30 s recordings at 100 Hz. *p*-values are from paired *t*-tests.

	*n*	VA-ECMO	Hemi-ECMO	*p*-Value
Mean Left Atrial Pressure		mmHg	mmHg	
2000 RPM	12	27.4 ± 0.75	32.1 ± 0.69	<0.001
2500 RPM	12	26.3 ± 1.34	26.6 ± 1.95	0.679
3000 RPM	12	23.9 ± 1.47	18.8 ± 1.65	<0.001
3500 RPM	12	26.9 ± 2.55	16.7 ± 2.71	<0.001
4000 RPM	12	28.2 ± 2.91	11.4 ± 2.84	<0.001
Aortic Ejection		L/min	L/min	
2000 RPM	12	0.67 ± 0.031	0.27 ± 0.058	<0.001
2500 RPM	12	0.15 ± 0.046	0.21 ± 0.046	0.333
3000 RPM	12	0.00 ± 0.002	0.14 ± 0.033	0.002
3500 RPM	12	0.00 ± 0.002	0.12 ± 0.037	0.009
4000 RPM	12	0.00 ± 0.001	0.08 ± 0.027	0.012
Pump Flow		L/min	L/min	
2000 RPM	12	1.38 ± 0.022	1.47 ± 0.024	<0.001
2500 RPM	12	2.08 ± 0.014	2.02 ± 0.036	0.036
3000 RPM	12	2.66 ± 0.027	2.56 ± 0.046	0.017
3500 RPM	12	3.25 ± 0.027	3.11 ± 0.054	0.003
4000 RPM	12	3.84 ± 0.036	3.69 ± 0.068	0.008
Coronary Artery Flow		mL/min	mL/min	
2000 RPM	12	90.3 ± 3.71	88.5 ± 3.41	0.302
2500 RPM	12	100.2 ± 3.26	74.3 ± 2.19	<0.001
3000 RPM	12	120.3 ± 4.73	57.5 ± 3.37	<0.001
3500 RPM	12	141.1 ± 5.07	54.6 ± 5.08	<0.001
4000 RPM	12	152.3 ± 3.71	56.6 ± 4.83	<0.001

## Data Availability

Data available upon request to corresponding author.

## Abbreviations

The following abbreviations are used in this manuscript:

VA-ECMO	Venoarterial extracorporeal membrane oxygenation
LV	Left ventricle
BVMCL	Biventricular mock circulatory loop
mLAP	Mean left atrial pressure
RPM	Revolutions per minute
LVF	Left ventricular failure
BVF	Biventricular failure
PVA	Pressure–volume area
